# Optimization of Ruthenium Dioxide Solid Contact in Ion-Selective Electrodes

**DOI:** 10.3390/membranes10080182

**Published:** 2020-08-09

**Authors:** Nikola Lenar, Beata Paczosa-Bator, Robert Piech

**Affiliations:** Faculty of Materials Science and Ceramics, Mickiewicza 30, AGH University of Science and Technology, PL-30059 Krakow, Poland; nlenar@agh.edu.pl (N.L.); rpiech@agh.edu.pl (R.P.)

**Keywords:** anhydrous and hydrous ruthenium dioxide, porous microstructure, high capacity, stable measuring signal

## Abstract

Ruthenium dioxide occurs in two morphologically varied structures: anhydrous and hydrous form; both of them were studied in the scope of this work and applied as mediation layers in ion-selective electrodes. The differences between the electrochemical properties of those two materials underlie their diverse structure and hydration properties, which was demonstrated in the paper. One of the main differences is the occurrence of structural water in RuO_2_•xH_2_O, which creates a large inner surface available for ion transport and was shown to be a favorable feature in the context of designing potentiometric sensors. Both materials were examined with SEM microscope, X-ray diffractometer, and contact angle microscope, and the results revealed that the hydrous form can be characterized as a porous structure with a smaller crystallite size and more hydrophobic properties contrary to the anhydrous form. Potentiometric and electrochemical tests carried out on designed GCD/RuO_2_/K^+^-ISM and GCD/RuO_2_•xH_2_O/K^+^-ISM electrodes proved that the loose porous microstructure with chemically bounded water, which is characteristic for the hydrous form, ensures the high electrical capacitance of electrodes (up to 1.2 mF) with consequently more stable potential (with the potential drift of 0.0015 mV/h) and a faster response (of a few seconds).

## 1. Introduction

Ruthenium dioxide can be considered as undeniably noteworthy amongst various electroactive materials previously used as mediation layers in solid-contact ion-selective electrodes (ISEs). One of the features characterizing solid-contact layer material is its electrical capacity, which is desired to be as high as possible to ensure the stable and fast potentiometric response of ISEs. As previously presented in [1,2], ruthenium dioxide allows achieving the highest capacitance of potassium-selective electrode with a polymeric membrane as a single-component layer (being conquered by hybrid layers consisting of two and more components). The success of ruthenium dioxide as a material for mediation layers in ion-selective electrodes lies in its porous structure and small grains providing high surface area and good charge-transfer characteristics [3,4].

In the literature, two main forms of the amorphous ruthenium dioxide, anhydrous (RuO_2_) and hydrous form (RuO_2_•xH_2_O), are mentioned and described [5,6,7,8,9]; both of them were studied in the scope of the presented work and implemented as mediation layers in potassium-selective electrodes.

The two materials (anhydrous and hydrous ruthenium dioxide) consist of primary grains clustered together in larger secondary grains. The size, packing, and surface area of these derived grains depend on the water content in the oxide’s structure [3]. Anhydrous RuO_2_ is characterized by grater and densely packed grains in comparison to hydrous ruthenium dioxide and its loosely packed minute grains, which ensure its higher surface area.

The difference in the structure of both materials is also based on the difference in the type of pores. In the structure of ruthenium dioxide, two types of pores that participate in charge transfer processes can be found and characterized separately—mesopores, obtained from secondary particles, and micropores. Macropores can be compared to the highways for the ion transport, while low-conducting micropores can be related to the busy city roads. Due to the packed structure of anhydrous RuO_2_, this type of oxide is low microporous, while in the loose morphology of hydrous RuO_2_, substantial hydrated macropores can be found [10]. Therefore, in anhydrous ruthenium dioxide, the conduction processes are connected only to the occurring macropores, while in the hydrous form, both types of pores are effectively used for the conduction.

The slow and controlled ion conduction via micropores is responsible for the low grain-boundary charging and leading to the increase of the capacitance value of the hydrous form in contrast to the hydrous one, which uses only easy accessible mesopores [11].

Hence, the decrease of water in ruthenium oxide’s structure that is chemically bound to the oxide’s grains is attributed to the increase of the grain size and decrease in micropores content, which leads to the decrease in ionic capacitance.

The overall capacitance of various morphologies of RuO_2_ is the totality of three partial capacitances C_dl_, C_ad_, and C_irr_, which are named the electric double layer capacitance, the adsorption related charge, and the irreversible redox related charge, respectively [3]. Whilst the values of the non-faradaic electrical double layer capacitance of both hydrous and anhydrous forms of RuO_2_ were found to be similar due to their comparable electrochemically active surface area, the values of C_ad_ and C_irr_ are varied and depend on the hydration degree. Hydrous oxide demonstrates larger C_ad_ values than its anhydrous form due to the large ion-diffusion occurring in hydrated nanoparticles yet smaller C_irr_ values, which can be attributed to the lack of grain-boundary charging. The measurements conducted in the scope of this work allowed comparing the total capacitance of both forms of ruthenium dioxide.

Another feature of ruthenium dioxide that has to be discussed is its charge-storage properties resulting from charge-transferred reactions between the oxide’s layer surface and electrolyte. In both hydrous and anhydrous form, those processes are initiated by the electrons and protons introduced to the material’s surface from electrolyte [2,12,13]. A detailed and suggested mechanism representing how RuO_2_ works as an ion-to-electron transducer was presented in previous work [1].

Contrary to the hydrous form, anhydrous RuO_2_ is reported to exhibit high electronic conductivity yet low proton mobility [14]. To increase the proton conductivity, structural water can be introduced into the rutile structure of ruthenium dioxide, as water film in-between grains allows both protons and electrons to be transported through the oxide’s layer [3]. Moreover, the porous structure of RuO_2_•xH_2_O ensures a shorter diffusion distance in contrast to the anhydrous form [11].

Taking into consideration all the mentioned features and differences between both studied materials, it can be concluded that hydrous ruthenium dioxide exhibits more favorable characteristics in the context of designing potentiometric sensors. Augustyn et al. [11] report that the high electrical capacitance and rapid faradaic reaction of RuO_2_•xH_2_O are induced by its unique features, such as rapid electron transport caused by the metallic conductivity of RuO_2_, rapid proton transport as a result of the presence of structural water, and the redox behavior of various Ru–RuO_x_ states allowing for faradaic energy storage and a large surface area.

The differences between both forms of RuO_2_ (hydrous and anhydrous) used for solid contact layers—those described in the Introduction and those presented in the Materials Characteristic section—have its reflection in the performance of ion-selective electrodes. Many different parameters such as the type of solvent, amount of oxide, hydration level, and layer thickness affect the electrical capacitance value. In order to help scientists who are concerned obtain similar results of capacitance for the ruthenium dioxide layer, in this article, we present a completely optimized procedure for the application of ruthenium oxide layers.

## 2. Experimental Section

### 2.1. Materials and Chemicals

Both studied forms of ruthenium dioxide—hydrous and anhydrous—were obtained commercially from Alfa Aesar (Haverhill, MA, USA) and Acros Organics (Fair Lawn, NJ, USA), respectively.

Potassium-selective membrane components—ionophore, lipophilic salt, plasticizer and polyvinyl chloride (PVC)—were purchased from Sigma Aldrich (St. Louis, MO, USA).

The organic solvents tetrahydrofuran (THF), dimethylformamide (DMF), and ethylene glycol (Gly) used for layer materials dispersion and membrane components dissolution were purchased from Sigma Aldrich.

Potassium Chloride KCl used to prepare standard potassium solutions was obtained from POCH (Gliwice, Poland). All materials were used as obtained without any further purification. For aqueous solutions preparation, distilled and deionized water was used.

### 2.2. Apparatus

Before being implemented onto electrodes, ruthenium dioxide (hydrous and anhydrous) was examined using a Scanning Electron Microscope (LEO 1530, Carl Zeiss, Wetzlar, Germany), contact angle microscope (Theta Lite with One Attension software by Biolin Scientific, Gothenburg, Sweden), differential scanning calorimeter (type DSC 2010, TA Instruments, New Castle, DE, USA), and X-ray diffractometer (X’Pert Pro with HighScore Plus software by PANalytical, Malvern, UK).

For the electrochemical tests carried on electrodes covered with studied layers of both types, the Autolab analyzer (Eco Chemie AUT32N.FRA2-AUTOLAB, ΩMetrohm, Herisau, Switzerland) analyzer) with NOVA software was used. All tests including cyclic voltammetry (CV), electrochemical impedance spectroscopy (EIS), and chronopotentiometry (Ch) were conducted in the 3-electrode cell consisting of a working electrode—a glassy carbon disc electrode covered with a RuO_2_ layer, reference electrode, and auxiliary electrode—a glassy carbon rod. All tests conducted with the use of an Autolab analyzer were performed with the use of 10^−1^ M KCl acting as electrolyte.

Designed ion-selective electrodes, after being carefully studied using the mentioned electrochemical techniques, were tested toward examining their potentiometric performance with use of the 16-channel Lawson Labs potentiometer for this purpose. The potential of all studied electrodes was measured against the reference electrode Ag, AgCl/3M KCl (ΩMetrohm, 6.0733.100 type) and double-junction electrode (ΩMetrohm, 6.0729.100 type) for long-lasting stability tests and in the presence of a platinum rod acting as an auxiliary electrode.

### 2.3. Electrodes Preparation

Before being casted with the studied layers and Ion Selective Membrane (ISM), the Glassy Carbon Disc (GCD) electrodes’ surface was prepared accordingly by polishing electrodes on alumina slurries (0.1 and 0.05 μm subsequently) and rinsing them with water and methanol.

In order to determine the difference between hydrous and anhydrous ruthenium dioxide in context of their influence on the ion-selective electrodes’ properties, three groups of electrodes were studied. The first group consists of three coated-disc electrodes (GCD/K^+^-ISM), which act as a control group providing the base results for modified RuO_2_-contacted electrodes.

The other two groups include solid-contact electrodes (three per group)—one group with hydrous RuO_2_ (GCD/RuO_2_•xH_2_O/K^+^-ISM), and one with anhydrous RuO_2_ (GCD/RuO_2_/K^+^-ISM) as the mediation layer.

The anhydrous RuO_2_ layer was prepared by dispersing 10 mg of RuO_2_ powder in 0.5 mL of DMF (which corresponds to the RuO_2_ concentration of 20 mg/mL) with the use of ultrasonic washer. The hydrous oxide’s layer was obtained analogously, and the concentration of RuO_2_•xH_2_O was 7 mg/mL DMF.

Both layers were implemented onto electrodes by topping them with 15 μL of layer solution using the simple and fast drop-casting method. After casting, electrodes were left until complete solvent evaporation in the elevated temperature (approximately 70 °C). After removing the DMF, solid particles of RuO_2_ remain on the electrode’s surface and adhere physically to the electrode material. No additional chemicals or stabilizers are used to attach the layer to the GCD electrode.

Afterwards, six electrodes with dried layers (three per type) and three electrodes without any layer (control electrodes) were covered twice with 30 μL of membrane solution which consisted of potassium ionophore (I) 1.10% (*w*/*w*), lipophilic salt (KTpClPB) 0.25% (*w*/*w*), plasticizer (o-NPOE) 65.65% (*w*/*w*), and PVC 33.00% (*w*/*w*) dissolved in tetrahydrofuran. Casted electrodes were left to dry at room temperature.

All prepared electrodes (12 items) were conditioned in 0.01 M K^+^ ion solution before being used for electrochemical measurements.

## 3. Results and Discussion

Before examining their suitability as mediation layers, both forms of ruthenium dioxide were subjected to tests in order to define their microstructure, wetting properties, crystallite size, and thermal behavior. All performed analysis enabled distinguishing and comparing the materials and helped reveal which of the material properties are crucial when applying them into potentiometric sensors.

### 3.1. Structure and Morphology Characterizations

SEM scans of both studied forms of ruthenium dioxide confirmed the differences mentioned in the introduction part. The grains of anhydrous ruthenium dioxide are significantly bigger in comparison comparing to those observed for the hydrous form. The hydrous RuO_2_ material is formed with minute particles, while in anhydrous oxide, grains of various sizes and aspect ratios can be found. 

**Figure 1:**
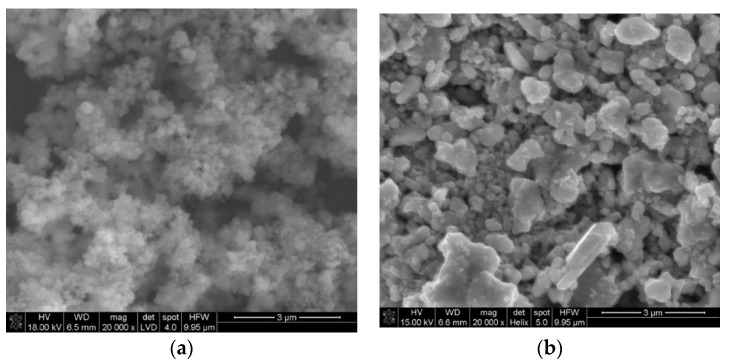
SEM scans of (**a**) hydrous RuO_2_ and (**b**) anhydrous RuO_2_.

As the grains observed on scans (Figure 1) are visibly agglomerated, to compare the size of single crystallites, the X-ray diffraction method was incorporated.

**Figure 2:**
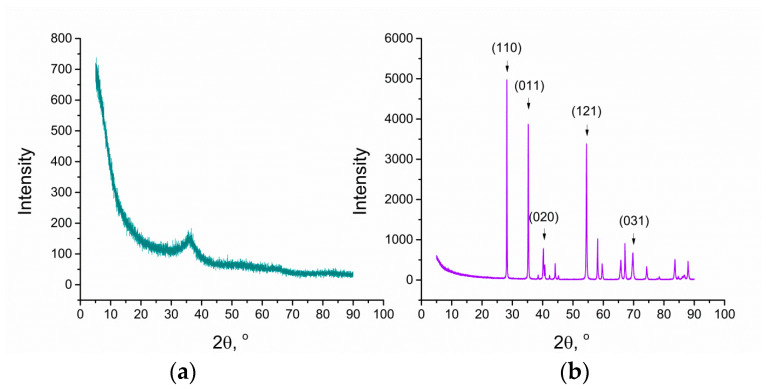
X-ray diffractogram of (**a**) hydrous RuO_2_ and (**b**) anhydrous RuO_2_.

The X-ray diffraction method was used for the structural characterization of both examined materials. The obtained X-ray diffraction patterns show differences between hydrous and anhydrous RuO_2_, indicating that the water content in its rutile structure decides the peak width and peak intensity. As known from the literature [15,16], the peak intensity decreases and the peak width increases with the increasing water content. The increase of the peak width can be attributed to the decrease in the primary particle size, and the decrease in peak intensity suggests a decrease in the amount of crystalline RuO_2_ and/or inferior crystallinity [17]. Based on the literature and obtained XRD patterns (Figure 2), it can be concluded that anhydrous RuO_2_ is more crystalline and is characterized by a greater crystallite size contrary to the hydrous form. Figure 2a shows the amorphous halo with no diffraction peaks, which suggests that the hydrous form of oxide is less crystalline.

The powder XRD pattern of the anhydrous RuO_2_ (Figure 2b) contains the diffraction peaks at 28.1°, 35.1°, 40.1°, 54.3°, and 69.6°, which can be indexed to (110), (011), (020), (121), and (031) reflections, respectively [18]. The detected phase of ruthenium dioxide belongs to the 2/mnm space group in the tetragonal crystal system.

Contrary to the one of hydrous form, the diffractogram obtained for the anhydrous ruthenium dioxide contains well-defined, sharp reflections; hence, it was possible to determine the size of the single crystallites assembling this material.

Crystallite size (D) was calculated according the Scherrer equation D = kλ/Bcosθ [19] based on the wavelength (λ), shape factor (k), peak position (given by θ), and its full-width at half-maximum (B), with the use of HighScore Plus software. The size calculated from the data for the five most intensive peaks was of 69.8 ± 4.9 nm.

### 3.2. Hydration Structure and Wettability

**Figure 3:**
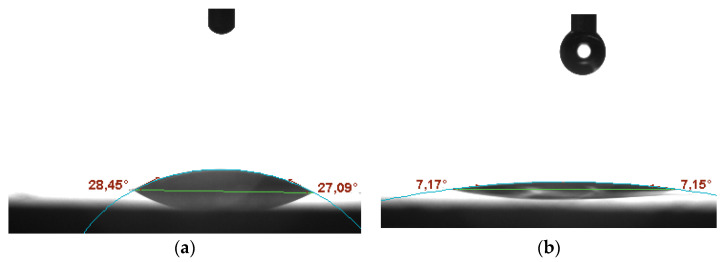
Contact angle microscope pictures of (**a**) anhydrous RuO_2_, (**b**) hydrous RuO_2_.

Contact angle microscope tests allowed examining the degree of wetting for the studied materials. A water drop was released onto the surface of hydrous and anhydrous ruthenium dioxide, and the angle between the materials’ surface and tangent to the formed water film was measured with the use of the microscope’s software. The obtained microscope pictures (Figure 3) display that both types of oxide can be characterized as hydrophilic with the contact angle of 28° for the anhydrous form (Figure 3a) and 7° for the hydrous form (Figure 3b).

**Figure 4:**
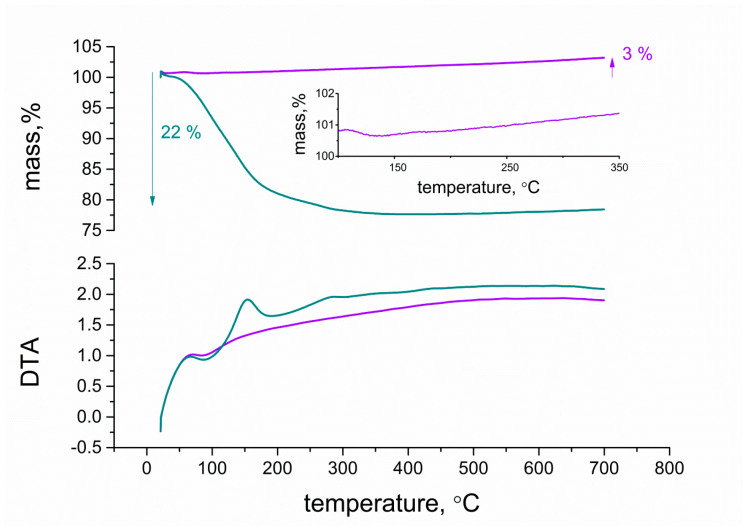
DTA/TG (Differential Thermal Analysis/Thermogravimetry) diagrams of anhydrous (violet curves) and hydrous (blue curves) RuO_2_ (upper curves: TG analysis, bottom curves: DTA).

In order to characterize and compare the structures of hydrous and anhydrous ruthenium dioxide more thoroughly, DTA/TG (Differential Thermal Analysis/Thermogravimetry) plots are presented in Figure 4. First, 11 mg samples of RuO_2_ and RuO_2_•H_2_O powder were examined for the purpose of this measurement, and the temperature increased at a 10 °C/min rate in the range from 0 to 700 °C.

Thermogravimetry analysis was performed in order to estimate the weight alterations during the course of measurement. For hydrous ruthenium dioxide, the weight decrease occurs continuously from 0 to 300 °C, and the amount of weight loss in ruthenium oxide was measured to be approximately 22% of sample mass. As described in the literature [19], firstly, the mass loss is associated to the desorption of physically adsorbed water and secondly to the desorption of the chemically bounded water contained in the oxide’s structure.

For anhydrous oxide, the weight firstly decreased, which could be attributed to the removal of impurities or water desorption from the surface of the oxide particles, and it eventually increased (3%) during the course of measurement. This phenomenon can be attributed to the oxidation process that transferred ruthenium IV into a higher oxidation state, which contributed to the weight gain of the sample mass [20].

The DTA results obtained for anhydrous RuO_2_ depicted one peak at ca. 100 °C associated with the removal of physically adsorbed water on the surface of nanoparticles. An analogous peak was observed for hydrous RuO_2_, which besides chemically bounded water, contains the same surface adsorbed water as the anhydrous one. On the DTA curve of hydrous ruthenium oxide nanoparticles, two more peaks were observed—at the temperatures near 150 °C and 300 °C, respectively. The first sharp peak, centered at ca. 150 °C, is attributed to the decomposition of chemically bounded water within nanoparticles. The second broad peak, at ca. 300 °C, is associated to the fusion and growth of ruthenium oxide nanoparticles to form larger crystals [19,21].

### 3.3. Electrochemical Capacitance Characterizations

Electrical parameters of solid-contact ISEs consisting of a mediation layer and ion-selective membrane can be considered as the result of values obtained for the membrane or layer itself. To learn about the properties of solid layers more thoroughly, layers can be studied after being implemented onto the glassy carbon electrode’s surface using such techniques as cyclic voltammetry, electrochemical impedance spectroscopy, and chronopotentiometry. All the mentioned techniques allow determining the most significant parameter, which is electrical capacitance; hence, the results can be easily compared.

#### 3.3.1. Cyclic Voltammetry Measurements

Firstly, electrical capacitance was determined using the cyclic voltammetry technique by cycling the potential of a working electrode and measuring its current response.

**Figure 5:**
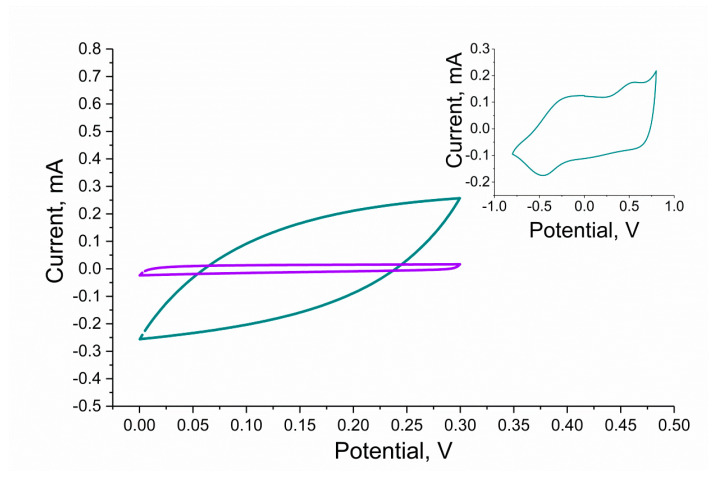
Comparison of electrochemical properties of anhydrous (violet curve) and hydrous (blue curve) RuO_2_ tested with the use of cyclic voltammetry in the potential range from 0 to 0.3 V, inset: voltammogram of hydrous RuO_2_ registered in a broader potential window from −1.0 to 1.0 V. Remaining cyclic voltammetry (CV) parameters: scan rate: 0.1 V/s, electrolyte: 0.01 M KCl.

RuO_2_-based layers were scanned with the speed of 0.1 V/s in the potential window between 0 and 0.3 V, and the CV curves for both studied materials were recorded and presented in Figure 5. The potential range was selected carefully so that no electrochemical reactions occur during the measurement and so the electrical capacitance value can be determined. The absolute current value observed in the middle of the potential window (that is, for the potential value of approximately 0.15 V) can be used for estimating the electrical capacity of the material, as divided by the scan rate (0.1 V/s), it gives the capacitance value. When comparing the curves recorded for the RuO_2_•xH_2_O and RuO_2_ layers, it can be observed that the first material is characterized by higher capacity than the other, as the current values are higher at the GCD/RuO_2_•xH_2_O voltammogram. The obtained results are presented in Table 1.

The inset to Figure 5 presents a voltammogram of hydrous ruthenium dioxide obtained using the same scanning speed yet a broader potential window (−1.0 to 1.0 V) in which it was possible to observe the peaks assigned to the ionic exchange processes and redox reactions as described by Majumdar et al. in [3].

#### 3.3.2. Electrochemical Impedance Spectroscopy Measurements

**Figure 6:**
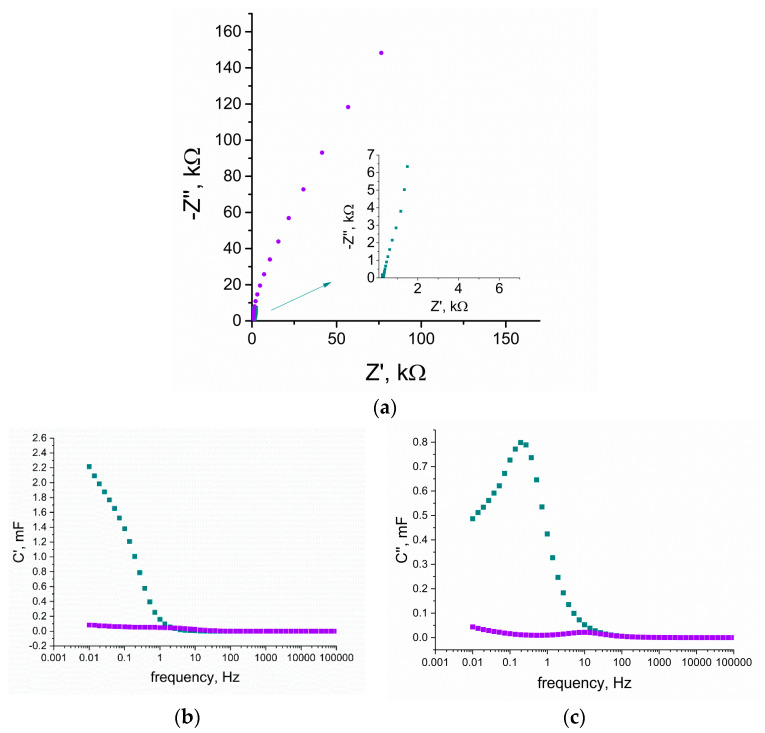
Comparison of electrochemical properties of anhydrous (violet curves) and hydrous (blue curves) RuO_2_ tested with the use of electrochemical impedance spectroscopy in the frequency range of 100 kHz and 0.01 Hz with an amplitude of 0.01 V and set potential value of 0.15 V in 0.01 M KCl as electrolyte, (**a**) electrochemical impedance spectroscopy (EIS) curves of both tested layers, inset: closer look at hydrous RuO_2_, (**b**) real part of capacitance (C’) change vs. frequency, (**c**) imaginary part of capacitance (C”) change vs. frequency.

The electrochemical impedance spectroscopy technique was also implemented onto the experiment to study the properties of the RuO_2_-based layers. The Nyquist plots recorded for both studied materials were compared and presented in Figure 6a. Materials were examined in the frequencies range between 100 and 0.01 Hz with an amplitude of 0.01 V and set potential value of 0.15 V.

For low frequencies, the capacitance (C) value can be calculated using the C = 1/(2πZf) equation, where f stands for the frequency (the lowest examined: f = 0.01 Hz) and Z stands for the imaginary part of the impedance. The obtained capacitance value was juxtaposed with the results from the other techniques in Table 1.

Using the real part of the capacitance versus frequency plot (Figure 6b), it can be seen that with the decrease in frequency, C’ increases sharply for the hydrous RuO_2_ material, contrary to the flat dependency observed for anhydrous RuO_2_. The capacitance reaches around 2.2 mF for RuO_2_•xH_2_O and 0.15 mF for RuO_2_, yet the whole capacitance is not reached (the value still increases with the decreasing frequency). This phenomena occurs because ions from the electrolyte have not reached the whole electrode’s material porosity.

Figure 6c presents the change of C” versus frequency. The imaginary part of the capacitance C’ goes through a maximum at a frequency f_0_, with a time constant defined as t_0_ = 1/f_0_ [22]. This time constant is described as a dielectric relaxation time that characterizes the whole system [23]. The relaxation time for hydrous RuO_2_ is 5 s and for anhydrous, it is 0.07 s.

#### 3.3.3. Chronopotentiometry

Electrical parameters, which can be determined with chronopotentiometry, such as capacitance and resistance, can be used to characterize both electroactive layers and electrodes with polymer membranes including those layers. To examine the layer itself, the electroactive material is dropped in form of the solution on the surface of the glassy carbon disc electrode and left until the solvent evaporation. Solid-contact and coated-disc electrodes are studied as obtained (according to the preparation procedure described in the work) without any modifications.

**Figure 7:**
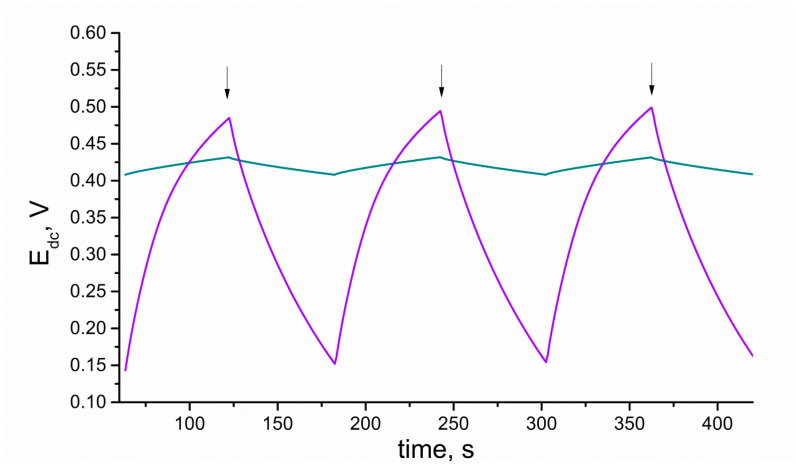
Comparison of electrochemical properties of anhydrous (violet) and hydrous (blue) RuO_2_ tested with the use of chronopotentiometry. Arrows point the moment of the current sign change.

**Figure 8:**
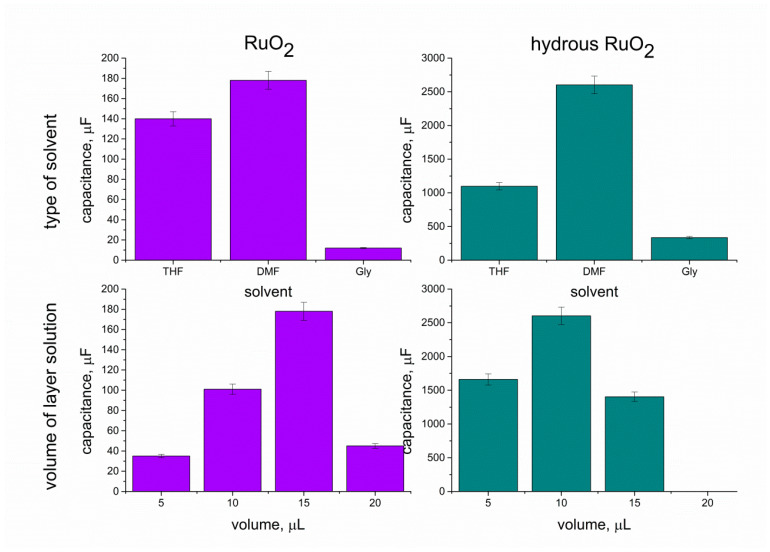
Capacitance values obtained for anhydrous (violet) and hydrous (blue) ruthenium dioxide dispersed in various solvents and applied in different amounts onto the electrode’s surface.

**Figure 9:**
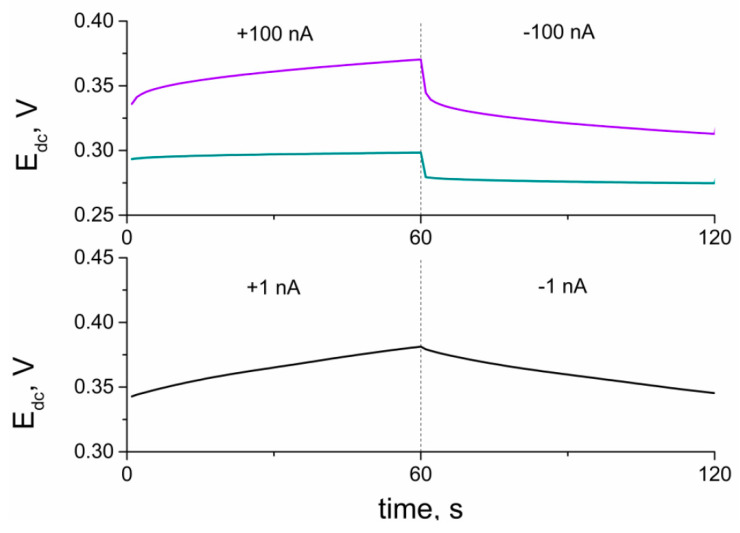
Part of chronopotentiograms recorded for solid contact electrodes: GCD/RuO_2_•xH_2_O/K^+^-ISM electrode (blue curve), GCD/RuO_2_/K^+^-ISM electrode (violet curve) and coated disc GCD/K^+^-ISM electrode (black curve).

Both layers and layers with membranes were examined using the same technique, and the results are presented in Figure 7, Figure 8 and Figure 9, respectively.

The electrical capacity of RuO_2_-based layers and further solid-contact electrodes (C) was determined using the chronopotentiometry technique. The principle of this electrochemical method underlies recording the potential of an electrode (E_dc_) while the current (I) is forced to flow through the 3-electrode cell (which consists of a working GCD electrode, reference electrode, and auxiliary electrode). The method was programmed for the time (t) of 60 s of +1 nA current flow followed by 60 s of −1 nA current flow. The program covered six steps and lasted 360 s in total. After every 60 s, the current sign changed, the potential jump (ΔE_dc_) is observed, and the potential is recorded for the following 60 s (with the opposite current sign). This method allows calculating the capacitance value using the mentioned parameters and the ΔE_dc_/Δt = I/C equation.

As the capacitance value of electrodes determines their analytical and electrical performance, this parameter was used to evaluate and compare layers of different types (form), thicknesses, or other varied factors. Therefore, the chronopotentiometry method was applied to select the best type amongst the studied materials, that is, the one of the highest capacitance value. The aim was to select the most favorable solvent for RuO_2_ particles and the most optimum volume of layer solution to be casted on the electrode, and eventually, to compare the electrical capacity of hydrous and anhydrous form of ruthenium dioxide. Tested solvents included THF, DMF, and ethylene glycol (Gly), and all of them were used to prepare solutions of hydrous and anhydrous forms of oxide. The capacitance values obtained for each solvent and layer volume were presented in columns and compared in Figure 8.

First, 10 μL of each solution was casted onto electrodes, and the capacitance values were as follows: 140 μF, 12 μF, and 101 μF for the THF-RuO_2_, Gly-RuO_2_, and DMF-RuO_2_ layers, respectively and 1098 μF, 336 μF, and 2602 μF for the THF-RuO_2_•xH_2_O, Gly-RuO_2_•xHO, and DMF-RuO_2_•xH_2_O layers. 

As presented, the highest capacitance value was obtained for both DMF-based studied layers, which allowed indicating this solvent as the most favorable in the context of designing solid-contact electrodes. Between the hydrous and anhydrous form of RuO_2_, there is a significant difference in the electrical capacity of both materials, and the hydrous form turned out to exhibit a much higher capacitance value. 

After selecting the right solvent, layers of different thicknesses were examined. DMF-based RuO_2_ and RuO_2_•xH_2_O solutions were dropped onto the electrodes surface in the following amounts: 5 μL, 10 μL, 15 μL, and 20 μL; the results were as presented: 35 μF, 101 μF, 178 μF, and 45 μF for anhydrous form and 1660 μF, 2602 μF, and 1404 μF for hydrous form. The layer of 20 μL was not examined in the case of hydrous form as after placing the electrode into the K^+^ ion solution, the RuO_2_•xH_2_O particles were detached from the electrode’s surface. The selected volume for RuO_2_ DMF-based layer solution was of l5 μL and for the hydrous form, it was of 10 μL.

The values of capacitance (for the optimized DMF-based layers) obtained using the chronopotentiometry technique were presented and compared with the other two applied electrochemical techniques (CV and EIS) in Table 1. The values of electrical capacitance obtained from all implemented techniques were in good agreement for both studied types of layers. The approximate capacitance value for the anhydrous RuO_2_ layer was of 0.2 mF, which is more than 10 times lower in comparison to the value achieved by the hydrous layer (approximately 2.5 mF). Those differences confirmed that the morphological and structural properties of the layers translate into their electrochemical characteristics.

When comparing the values presented in Table 1 (for layers) and those from the last column in Table 2 (for designed ion-selective electrodes), it can be seen that the properties of the layers strongly determine the properties of the designed electrodes. The differences observed between the RuO_2_ and RuO_2_•xH_2_O layers can be also recognized in between GCD/RuO_2_•xH_2_O/K^+^-ISM and GCD/RuO_2_/K^+^-ISM electrodes. Implementing the RuO_2_•xH_2_O layer of high electrical capacity allowed obtaining electrodes characterized by considerably high capacitance, while the capacitance of the GCD/RuO_2_/K^+^-ISM electrodes turned out to be more than 10 times smaller.

### 3.4. Electrical Parameters of Electrodes

#### 3.4.1. Chronopotentiometry

With the use of the same technique (chronopotentiometry) and the same parameters that need to be recorded to obtain the capacitance value, another two important electrical parameters can be determined: resistance and potential drift.

While it is desired for the electrical capacitance to be of the highest value, the resistance and potential drift are expected to be as low as possible. The resistance value can be calculated using potential jump (ΔE_dc_) and current (I) values with the use of the R_total_ = ΔE_dc_/2I equation. Potential drift is defined as the potential change divided by the time change, which makes the simple equation: ΔE_dc_/Δt.

All the electrical parameters’ values were presented in the table as averaged values calculated from results obtained for 3 electrodes from each group along with their standard deviations.

As presented, electrodes with a layer of hydrous RuO_2_ exhibited the highest electrical capacity, and consequently, the lowest resistance and potential drift of all tested groups. Electrodes from the GCD/RuO_2_/K+-ISM group, which are based on anhydrous oxide, presented considerably lower capacitance value and higher values of other electrical parameters. As mentioned in the Introduction, one of the differences between those two materials is the occurrence of structural water in RuO_2_•xH_2_O, which creates a large inner surface available for ion transport [11]. The total capacitance value of the hydrous form of oxide compared to the anhydrous one turned out to be much higher; therefore, the hydrous layer is more favorable in context of designing ion-selective electrodes. 

The results obtained using the Autolab analyzer allowed characterizing layers before their implementation onto electrodes (Figure 7) and further characterizing electrodes (Figure 9) before using them in potentiometric tests and experiments.

#### 3.4.2. Electrical Impedance Spectroscopy

**Figure 10:**
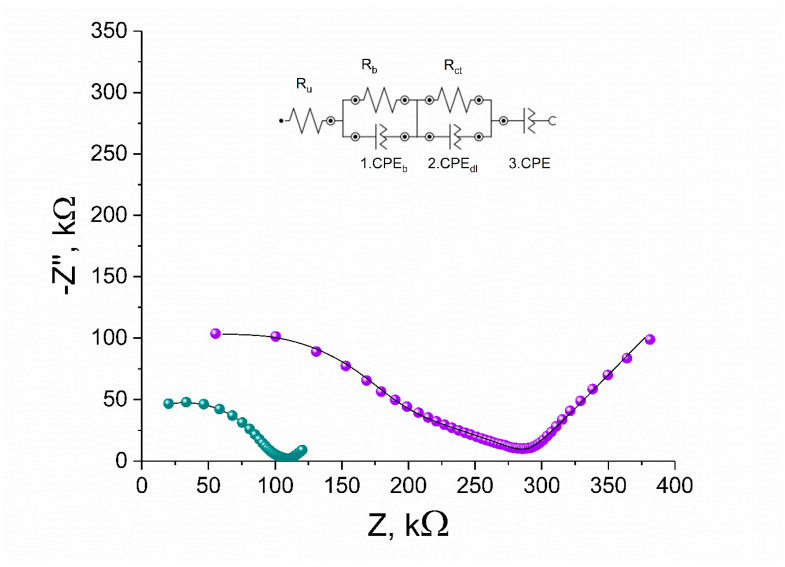
Impedance spectrum of GCD/RuO_2_•xH_2_O/K^+^-ISM (blue) and GCD/RuO_2_/K^+^-ISM (violet) electrode in 0.01 M KCl solution and equivalent electrical circuits. Frequency range: 100 kHz–0.01 Hz. Equivalent circuits are shown as insets (solid lines represent data fits).

Here, Electrical Impedance Spectroscopy was implemented again in order to determine the electrical parameters of RuO_2_-based electrodes covered with an ion-selective polymeric membrane. Impedance data were collected in the frequency range from 100 kHz to 0.01 Hz using an amplitude of 10 mV superimposed onto open-circuit potential (OCP). The EIS plots were presented in Figure 10 together with the equivalent circuit. The solid line represents the EIS data fitted to obtained EIS results using NOVA 2.1.4 software. The diameter of a semicircle in the high-frequency region represents the bulk membrane resistance R_b_, which for the GCD/RuO_2_•xH_2_O/K^+^-ISM electrode is 105 kΩ, and for the GCD/RuO_2_/K^+^-ISM electrode, it is 269 kΩ. The bulk resistance R_b_ results from the resistance of the ion-selective membrane and the resistance of the ISM inside the porous structure of the mediation layer; therefore, the resistance of the electrode with more porous material such as hydrous RuO_2_ is lower. In the low-frequency region, the EIS curve results from double-layer capacitance (CPE_dl_) and charge-transfer resistance (R_ct_), which provides the information about the ease of electron/ion transfer at the electrode interface. As presented in Table 3, an electrode with hydrous RuO_2_ as the mediation layer is characterized with lower resistance and higher capacitance (R_ct_ = 24 kΩ, CPE = 1106^(0.443)^ µS^(N)^ for the GCD/RuO_2_•xH_2_O/K^+^-ISM electrode) in contrast to the GCD/RuO_2_/K^+^-ISM electrode, for which R_ct_ = 128 kΩ and CPE = 185^(0.702)^ µS^(N)^. Therefore, it can be concluded that hydrous RuO_2_ allows fast charge transfer processes and guarantees a stable potential response of designed ISEs.

The bulk resistance and double-layer capacitance obtained with the use of EIS are comparable to the electrical parameters (resistance and capacitance) of studied electrodes evaluated using the Chronopotentiometry method.

### 3.5. Potentiometric Tests

Designed electrodes after being tested with the use of various electrochemical techniques, where the current value was a significant value, were implemented into the traditional potentiometric measurement. All studied groups, including two groups of solid-contact electrodes (GCD/RuO_2_/K^+^-ISM and GCD/RuO_2_•xH_2_O/K^+^-ISM) and a group of coated-disc electrodes (GCD/K^+^-ISM), were connected with potentiometer and placed into the measuring vessel together with reference and auxiliary electrodes in order to study their behavior in the conditions of typical potentiometric measurement (with no current presence).

Since the desired application of the presented sensors is the measurement of potassium content in the water samples, firstly, the sensors must have been characterized and their parameters strictly determined. Electrodes were examined with the use of the potentiometry method and defined by indicating the slope of the calibration curve and the standard potential.

**Figure 11:**
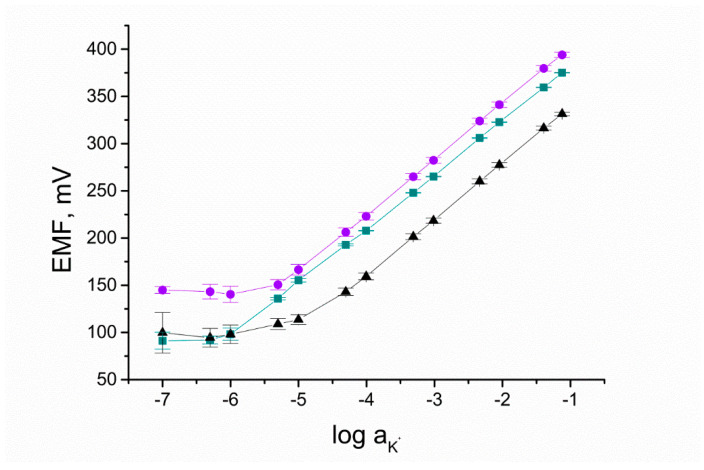
Calibration curves recorded for GCD/RuO_2_•xH_2_O/K^+^-ISM (■), GCD/RuO_2_/K^+^-ISM (●), and GCD/K^+^-ISM (▲) electrode.

The calibration curves are presented in Figure 11. As shown, the detection limit for the GCD/RuO_2_•xH_2_O/K^+^-ISM group is the lowest and reaches 10^−6^ M K^+^. Implementing anhydrous RuO_2_ into electrodes allowed obtaining a detection limit of 5•10^−5.5^ M K^+^. Both forms of ruthenium dioxide were found to improve the linear range of sensors, as for the coated-disc electrodes, the detection limit was only 10^−5^ M.

Based on the parameters of curves recorded in the function of Electromotive Force and logarithm of activity of K^+^, the equations were fixed for each group:

E = 57.37log a_K_^+^ + 439 [mV] for CGD/RuO_2_•xH_2_O/K^+^-ISM group (1)

E = 59.44log a_K_^+^ + 462 [mV] for CGD/RuO_2_/K^+^-ISM group (2)

E = 57.01log a_K_^+^ + 394 [mV] for GCD/K^+^-ISM group. (3)

With the use of designed sensors and presented equivalences, it is possible to estimate the potassium content based on the measured potential value. 

Comparing the convergence of results within a group of electrodes, it was possible to fix the average parameters for each group and determine the repeatability of the potential response. Based on the error bars presented in Figure 10, which correspond to the values of standard deviations calculated for the studied electrodes, it can be seen that the best compatibility between results was observed for the GCD/RuO_2_•xH_2_O/K^+^-ISM group.

What should be emphasized here is that amongst the solid-contact potassium selective electrodes presented in the literature (a select few are presented in Table 4), RuO_2_•xH_2_O-based electrodes exhibit satisfying analytical parameters such as theoretical Nernstian response in the wide K^+^ ions concentration range, moderate potential drift, and considerably high electrical capacitance. Therefore, it can be concluded that hydrous ruthenium dioxide applied alone and without any modifications allows obtaining the ion-selective electrode of parameters that are comparable to the best potentiometric methods described in the literature.

### 3.6. Stability of Response

In the addition to the presented chronopotentiometric results was the potential drift, which was calculated based on the value of the potentiometric response and forced current flow; herein, we present the stability of the designed sensors in terms of the traditional potentiometric measurement. The potential was recorded over the time of 21 h, and the potential drift was estimated based on the obtained results.

The stability of electrodes given by the potential/time ratio was as follows: 0.0015 mV/h for the GCD/RuO_2_•xH_2_O/K^+^-ISM electrode, 0.042 mV/h for the GCD/RuO_2_/K^+^-ISM electrode, and 0.48 mV/h for the coated-disc electrode. The results obtained at this stage are consistent with those of chronopotentiometry, as the electrical capacitance, which decides the electrodes’ ability to exhibit a stable potentiometric response, was of the highest value for sensors with an RuO_2_•xH_2_O mediation layer.

**Figure 12:**
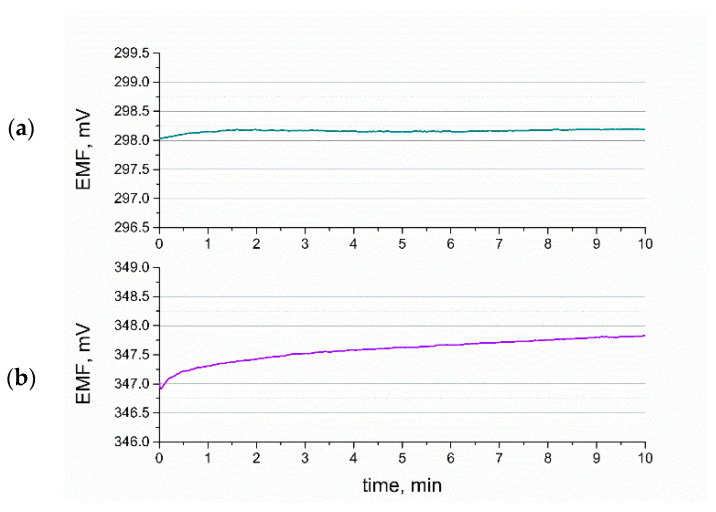
Potentiometric response of (**a**) GCD/RuO_2_•xH_2_O/K^+^-ISM electrode and (**b**) GCD/RuO_2_/K^+^-ISM electrode during first 10 min of measurement.

Figure 12 presents the potential response curves, which reflect the response time of the fabricated ISEs. The exemplary potential stability of the GCD/RuO_2_•xH_2_O/K^+^-ISM and GCD/RuO_2_/K^+^-ISM electrodes measured in a 0.01 M KCl solution during the first 10 min of measurement is presented in Figure 12a,b, respectively.

According to the IUPAC (International Union of Pure and Applied Chemistry) nomenclature for ion-selective electrodes, the time of response is equal to the time period between contacting the analyte and reaching 95% of the equilibrium potential value. As shown, the time of response for a hydrous RuO_2_-based electrode is as short as a few seconds, and the stable potential value is reached almost immediately. For the RuO_2_-based electrode, the equilibrium potential is reached after a few minutes (a 95% potential value is reached immediately), and the potential stability is worse in comparison with the GCD/RuO_2_•xH_2_O/K^+^-ISM electrode. 

### 3.7. Water Layer Test

**Figure 13:**
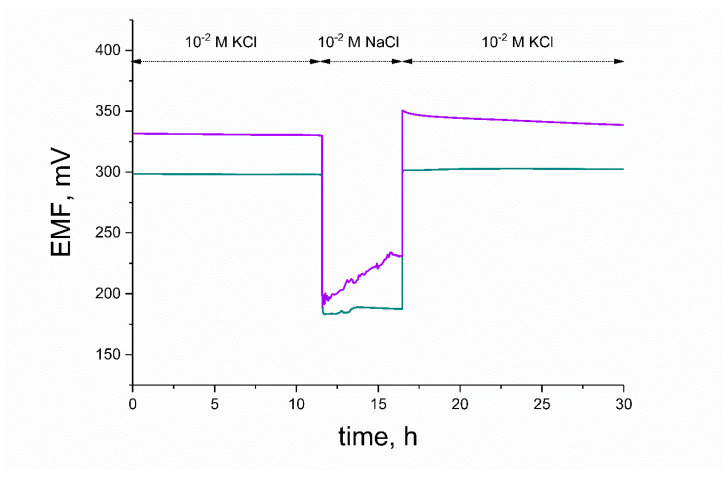
Water layer test of GCD/RuO_2_•xH_2_O/K^+^-ISM (blue curve) and GCD/RuO_2_/K^+^-ISM (violet curve) electrode.

The water layer test was conducted to examine the water uptake of studied electrodes during the course of potentiometric measurement. Water from measured samples and conditioning solution tends to penetrate the membrane and form the thin film between the polymeric membrane and electronic conductor. This process may cause the potential drift of the electrodes’ response and result in deterioration of the membrane’s adherence; therefore, it is desired to eliminate the water layer. Water uptake can be limited by introducing the mediation layers that will prevent the accumulation of water. The ability of the studied ruthenium dioxide layers to eliminate the water layer was evaluated during a water layer test when exchanging primary ion (potassium) solution into sodium ion solution. Afterwards, the electrodes were placed into 10^−2^ M KCl solution for about 10 h, KCl was exchanged into 10^−2^ M NaCl solution to examine the potential drift, and after 5 h, it was exchanged back to the primary ion solution to examine the stability of potentiometric response. The potentiometric response was recorded with time, and the results are presented in Figure 13.

As shown, the response of the hydrous RuO_2_-based electrode was stable after contacting NaCl solution and the characteristic potential drift was not observed, contrary to the response of the electrode with an anhydrous layer. After being placed back into primary ion solution, the GCD/RuO_2_/K^+^-ISM electrode exhibited a substantial drift of potentiometric response, and the original equilibrium for K^+^ ions was established again after a few hours. The difference in behavior of two studied electrodes can be explained by the participation of structural water in the ion-exchange processes.

## 4. Conclusions

The differences between the hydrous and anhydrous form of ruthenium dioxide can be noticed at the very beginning of material characterization—the loose and porous microstructure of hydrous form with small crystallites and chemically bounded water is in opposition to the densely packed, macroporous structure of the anhydrous form. All the tested features of the studied materials—microstructure, wettability, and crystallite size—contribute significantly to the properties of materials as mediation layers in ion-selective electrodes. The small size of ruthenium dioxide particles ensured a high surface area, and the presence of the water in between grains provided extra pathways for ion transport; therefore, hydrous ruthenium dioxide turned out to be a more favorable material in the context of designing ion-selective electrodes.

This claim is supported by the results from chronopotentiometry, which revealed the high value of the electric capacitance and potentiometry. Potentiometric tests proved that the analytical performance of RuO_2_-based electrodes is considerably better compared to that of a non-modified coated-disc electrode. Between the hydrous and anhydrous ruthenium dioxide layers, we selected RuO_2_•xH_2_O-based electrodes as the most robust, with their wider linear range (up to 10^−6^ M K^+^) and stable potentiometric response (with the potential drift for this type of electrode of 0.0015 mV/h).

## Figures and Tables

**Table 1:** Electrical capacitance of layers (n = 3).

Group of Electrodes	Cyclic Voltammetry C ± SD [mF]	Electrochemical Impedance Spectroscopy C ± SD [mF]	Chronopotentiometry C ± SD [mF]
GCD/RuO_2_	0.13 ± 0.01	0.27 ± 0.02	0.18 ± 0.01
GCD/RuO_2_•xH_2_O	2.5 ± 0.1	2.3 ± 0.1	2.6 ± 0.1

**Table 2:** Electrical parameters of studied electrodes (n = 3). GCD: glassy carbon disc.

Group of Electrodes	Resistance ± SD [kΩ]	Potential Drift ± SD [μV/s]	Capacitance ± SD [μF]
GCD/K^+^-ISM	1022 ± 21	598 ± 20	1.67 ± 0.06
GCD/RuO_2_/K^+^-ISM	279.5 ± 0.5	532.8 ± 0.6	188 ± 2
GCD/RuO_2_•xH_2_O/K^+^-ISM	94.64 ± 0.07	81.1 ± 0.9	1233 ± 14

**Table 3:** Electrical parameters of studied electrodes.

Electrode Type	R_u_ (kΩ)	R_b_ (kΩ)	CPE_b_ (pS)^(N)^	R_ct_ (kΩ)	CPE_dl_ (nS)^(N)^	CPE (µS)^(N)^
GCD/RuO_2_•xH_2_O/K^+^-ISM	−32	105	34.2^(0.93)^	24	968^(0.294)^	1106^(0.443)^
GCD/RuO_2_/K^+^-ISM	−48	269	26.1^(0.87)^	128	117^(0.409)^	185^(0.702)^

**Table 4:** Electrical and potentiometric parameters compared for GCD/SC/K^+^-ISM electrodes with various materials applied as solid-contact (SC) layers: TCNQ—Tetracyanoquinodimethane; GR—Graphene; PEDOT(CNT)—Poly (3,4-ethylenedioxythiophene)–Carbon Nanotubes; MoO_2_—Molybdenum Dioxide; CIM—Colloid-Imprinted Mesoporous Carbon; CB-GR-FP—Carbon Black–Graphene–Fluorinated acrylic copolymer.

Electrode	Capacitance Value [μF]	Potential Drift [μV/s]	Linear Range [M]	Slope [mV/dec]	Reference
GCD/TCNQ/K^+^-ISM	132	12	10^−1^–10^−6.5^	58.68	[24]
GCD/GR/K^+^-ISM	91	12	10^−1^–10^−4.5^	59.2	[25]
GCD/PEDOT(CNT)/K^+^-ISM	83	12	10^−1^–10^−6^	57.7	[26]
GCD/MoO_2_/K^+^-ISM	86	11.67	10^−3^–10^−5^	55.0	[27]
GCD/CIM/K^+^-ISM	1000	1	10^−1^–10^−5.2^	59.5	[28]
GCD/CB-GR-FP/K^+^-ISM	1471	0.68	10^−1^–10^−6.5^	59.10	[29]
GCD/RuO_2_/K^+^-ISM	188	533	10^−1^–10^−5.5^	59.44	this work
GCD/RuO_2_●xH_2_O/K^+^-ISM	1233	81	10^−1^–10^−6^	57.37	this work
